# The Open Knowledge Foundation: Open Data Means Better Science

**DOI:** 10.1371/journal.pbio.1001195

**Published:** 2011-12-06

**Authors:** Jennifer C. Molloy

**Affiliations:** Department of Zoology, University of Oxford, Oxford, United Kingdom

## Abstract

Open data leads to better science, but overcoming the barriers to widespread publication and availability of open scientific data requires a community effort. The Open Knowledge Foundation Open Data in Science Working Group describes their role in this movement.

Data provides the evidence for the published body of scientific knowledge, which is the foundation for all scientific progress. The more data is made openly available in a useful manner, the greater the level of transparency and reproducibility and hence the more efficient the scientific process becomes, to the benefit of society. This viewpoint is becoming mainstream among many funders, publishers, scientists, and other stakeholders in research, but barriers to achieving widespread publication of open data remain. The Open Data in Science working group at the Open Knowledge Foundation is a community that works to develop tools, applications, datasets, and guidelines to promote the open sharing of scientific data. This article focuses on the Open Knowledge Definition and the Panton Principles for Open Data in Science. We also discuss some of the tools the group has developed to facilitate the generation and use of open data and the potential uses that we hope will encourage further movement towards an open scientific knowledge commons.

## Introduction

Science is built on data: its collection, analysis, publication, reanalysis, critique, and reuse. However, the current system of scientific publishing works against maximum dissemination of the scientific data underlying publications. Barriers include inability to access data, restrictions on usage applied by publishers or data providers, and publication of data that is difficult to reuse, for example, because it is poorly annotated or “hidden” in unmodifiable tables like PDF documents. In addition, there is a cultural reluctance to publish data openly, for multiple reasons—from researchers' fears about releasing data “into the wild” where they lack control over its usage to a lack of incentive or credit for doing so.

In response to these problems, multiple individuals, groups, and organisations are involved in a major movement to reform the process of scientific communication. The promotion of open access and open data and the development of platforms that reduce the cost and difficulty of data handling play a principal role in this.

One such organisation is the Working Group on Open Data in Science (also known as the Open Science Working Group) at the Open Knowledge Foundation (OKF). The OKF is a community-based organisation that promotes open knowledge, which encompasses open data, free culture, the public domain, and other areas of the knowledge commons. Founded in 2004, the organisation has grown into an international network of communities that develop tools, applications, and guidelines enabling the opening up of data, and subsequently the discovery and use of that data. Its working groups are in fields as broad as government, development, science, economics, archaeology, and geodata. However, all are united by the same organisational values and principles, and share a common understanding of openness, as set out in the Open Knowledge Definition (OKD; http://www.opendefinition.org/okd/).

The OKF Working Group on Open Data in Science (http://science.okfn.org/About/) began in 2009 with the purpose of developing guidelines, tools, and applications to promote open data in the sciences and enable scientists to maximise the use and impact of that data. It is now a diverse and international community of scientists, data wranglers, lawyers, and other individuals with interests in both open data and the broader concept of open science.

## The Open Knowledge Definition

The definition of “open”, crystallised in the OKD, means the freedom to use, reuse, and redistribute without restrictions beyond a requirement for attribution and share-alike. Any further restrictions make an item closed knowledge. It also emphasises the importance of usability and access to the entire dataset or knowledge work:

“The work shall be available as a whole and at no more than a reasonable reproduction cost, preferably downloading via the Internet without charge. The work must also be available in a convenient and modifiable form.”

**Figure 1:**
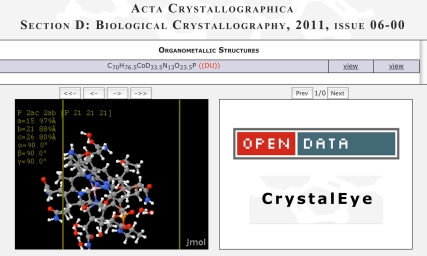
Screenshot of the CrystalEye entry for the structure of coenzyme cob(II)alamin with a copy of the OKF Open Data button displayed on the site.

This is an important consideration for scientific data where in some cases data is accessible, for example, in online supplements to published papers, but is not licensed to be reuseable; or it's accessible and reuseable but in a form that inhibits capture and modification. Prior to online supplementary materials, requesting and obtaining permissions and data was an extremely time-consuming process, but even with instant downloads, deciding what rights one has to reuse data can be confusing due to a lack of licensing and clear terms of use. In some cases, the supplementary data associated with papers is open even if the article itself is not; but this is often not explicit. Clear labelling and licensing is vital to save scientists the many hours they may spend discovering the openness or otherwise of datasets and becomes even more imperative as computerised analysis of the scientific literature increases, for example via data and text mining. Websites such as the crystallography data aggregator CrystalEye (http://wwmm.ch.cam.ac.uk/crystaleye/) prominently display an Open Data web button on their website and link to the Public Domain Dedication and License (PDDL) license as well as the OKD (Figure 1).

Deciding what constitutes open is particularly pertinent to the movement in science towards open access, or OA, which is related to open data but has different immediate goals. OA is defined in the Bethesda Statement (http://www.earlham.edu/~peters/fos/bethesda.htm) in terms that embrace open data. However, non-OA publishers often use the term to mean “free” access to publications. An important distinction is drawn within the open community between libre “free as in freedom”, as expressed in the OKD, and gratis “free as in beer”. The majority of OA journals appear to be gratis rather than libre—as of August 2011 only 1,549 (22%) of the 6,922 journals in the Directory of Open Access Journals (DOAJ) were licensed under Creative Commons, and some of these licenses contained non-commercial or non-derivative clauses. Therefore, the reader may not be free to do what they wish with the text or data as per the OKD.

To reduce confusion about what open data should look like, there was a need to extend the OKD with a new set of principles specific to the scientific field.

## The Panton Principles for Open Data in Science

In collaboration with John Wilbanks of Creative Commons, key members of the OKF—Rufus Pollock (University of Cambridge), Peter Murray-Rust (University of Cambridge), and Cameron Neylon (STFC)—spent two years developing a set of principles for publishing open scientific data, using the OKD and the Science Commons' Protocol for Implementing Open Access Data (http://sciencecommons.org/projects/publishing/open-access-data-protocol/) as precedents and guides. The result was the Panton Principles (see Box 1; http://www.pantonprinciples.org/), named after the Panton Arms pub in Cambridge where the majority of the drafting sessions occurred. The principles were officially launched in February 2010 and have since gained more than 150 endorsers.

**Box 1. Panton Principles in Summary**

1. When publishing data, make an explicit and robust statement of your wishes.
2. Use a recognised copyright waiver or license that is appropriate for data.
3. If you want your data to be effectively used and added to by others, it should be open as defined by the Open Knowledge/Data Definition—in particular, non-commercial and other restrictive clauses should not be used.
4. Explicit dedication of data underlying published science into the public domain via PDDL (http://opendatacommons.org/licenses/pddl/1-0/) or CCZero (http://creativecommons.org/publicdomain/zero/1.0/) is strongly recommended and ensures compliance with both the Science Commons Protocol for Implementing Open Access Data and the Open Knowledge/Data Definition.

The scope of the principles covers all primary experimental data published within or alongside research papers, including the data content of any table or graph and all images, audio, or video acting as the primary mechanism of data capture, e.g., protein gels or animal vocalisation recordings. The crux of the Panton message is that all such data—with very few exceptions—should be placed explicitly in the public domain. Good reasons for not releasing data would include the risk of violating patient privacy or revealing the precise location of an endangered species.

## The Open Data Movement in Science

The Panton Principles are not an isolated initiative but part of a wider movement to promote open data in science that is gathering momentum. Historically, scientific data has not been openly available, for a great variety of reasons. Some are technological—paper is not an efficient form of sharing datasets—but the web has opened up not just new possibilities for sharing, collaboration, and analysis, but also for exploring new forms of scientific enquiry. For example, automated text and data mining of large swathes of the published corpus of scientific knowledge is now feasible if such material is accessible.

Encouraging scientists to share their data is a challenge, even when it directly supports published work. A 2009 report by the Research Information Network [1] found that some researchers were unwilling to share their data openly due to fears of exploitation, particularly for datasets where they felt they could extract multiple publications; another problem is the lack of career rewards, recognition, or incentives to publish data, which makes it difficult for researchers to justify the time and effort required to make data available.

However, there is top-down pressure to move towards open data publication from funders such as the Wellcome Trust and the United Kingdom Research Councils as well as the United States National Institutes of Health (NIH), which published a joint statement to that end in February 2011 [2]. The European Commission and the Royal Society are both leading major enquiries into the future of the communication of scientific information, with reports due later this year. Open data in science has even appeared on government agendas; a recent report from the UK House of Commons Select Committee on Science and Technology examined research integrity and the peer review process and concluded that:

“Access to data is fundamental if researchers are to reproduce, verify and build on results that are reported in the literature … The presumption must be that, unless there is a strong reason otherwise, data should be fully disclosed and made publicly available. In line with this principle, where possible, data associated with all publicly funded research should be made widely and freely available…The work of researchers who expend time and effort adding value to their data, to make it usable by others, should be acknowledged as a valuable part of their role” [3].

Implementing open data more widely necessitates new infrastructure to support data archiving, as well as a change to how data fits within scientific publishing. Major OA publishers and their non-OA colleagues are joining forces to discuss these issues through groups like the Publishing Open Data Working Group led by BioMedCentral (BMC). Some journals are participating in a Joint Data Archiving Policy (JDAP), which requires deposition of data underlying papers in appropriate public repositories such as Dryad (http://datadryad.org/). Alternatively, direct publishing of data as a peer-reviewed “data paper” is now possible in the fields of biodiversity (http://www.gbif.org/; [4]) and ecology and environmental science (http://www.pangaea.de/ and http://www.earth-system-science-data.net).

There is also a role for individuals and communities to drive the open data message forward. Veli Vikberg, David R. Smith, and Jean-Luc Boevé won the 2011 BMC Open Data Award for their efforts in publishing the full ecological background data associated with a paper on the ecological phylogenetics of plant-feeding insects [5], which was above and beyond the DNA sequences that are the norm for such publications. Vikberg admitted that “credit…must go to a persistent, anonymous referee …who demanded—twice—that we also publish the background data” [6].

A single individual's persistence led to the open publication of data that would otherwise have been more difficult for researchers to obtain, which Vikberg acknowledges will aid reanalysis as new and improved models emerge in the ecological phylogenetics field. In addition, the research team gained recognition and reward from BMC and the members of the Open Data in Science working group on the judging panel.

Networks such as the OKF working group and other open data initiatives can play an important role in bringing enthusiastic individuals together to effect change. Further to encouraging researchers to publish data openly, we are dedicated to developing practical assistance in the form of tools and applications via our community of scientists who provide the problems and suggest possible solutions, and the developers who build them.

## Is It Open Data?

Requesting data from other researchers can be a tortuous and sometimes fruitless process. In a 2006 survey, 50.8% of US researchers reported that data withholding had exerted a negative effect on the progress of their research [7]. This problem could be overcome by sharing data freely online, but as discussed previously, discovering the terms of use of data can be a difficult and time-consuming task as this information is often not explicitly stated at the point of data viewing or download.

With this in mind, one of the first tools that the Open Data in Science working group created was “Is It Open Data?” (IIOD?; http://www.isitopendata.org/), a web application based on civil society websites such as What Do They Know? (WDTK?; http://www.whatdotheyknow.com). WDTK? allows users to make Freedom of Information requests for public sector or government information in the UK and records the resulting correspondence as a permanent and visible record in the public domain. In much the same way, IIOD? enables interested parties to request the open or closed status of data and data licensing details from providers such as academic publishers, research organisations, nongovernmental organisations, and all others making data available online.

It has already been used to contact major scientific journal publishers regarding the status of data in the supplementary documentation associated with published papers, and we would encourage others to contact their own journals of choice where data policies are unclear. In our first round of enquiries, the openness of data in Public Library of Science (PLoS) and BMC publications was confirmed, while Nature Publishing Group also stated that raw data extracted from their publications may be used as open data, with limited caveats. Over time, extensive and systematic requests to journals and other data providers are expected to build up a collection of position statements on data reuse that are currently unavailable without searching through the journal or publisher's websites individually. We hope this will result in fewer duplicated requests and save researchers valuable time.

## What the Reuse of Open Data Might Achieve

There is little point in opening up data if it is not used; it does not intrinsically lead to better science in and of itself, although it could be argued that the open publication of datasets will directly discourage fraud. It would be useful to evaluate the reuse of current open data, but evidence is limited due to issues in tracking data citations. However, it does appear that publicly sharing your data increases citation rate, at least in cancer microarray experiments [8], which is positive encouragement that open biological data is being reused. Evidence is also emerging that data archiving leads to an impressive scientific return per research dollar [9], which corroborates the obvious benefits of shared data in established databases such as GenBank and the Protein Data Bank (PDB) that have had such a huge impact on the biological field. To maximise this discovery and reuse, tools are required to assist in locating open data and making it usable, for example, extracting data from unmodifiable formats like PDF.

A current collaboration between the Open Data in Science working group, the Joint Information Services Council (JISC) funded DevCSI project, and Semantic Web Applications and Tools for Life Sciences (SWAT4LS) is a free workshop to generate semantic tools for the biological sciences (http://www.ukoln.ac.uk/events/devcsi/life-sciences-hackdays/index.html). As part of this we hope to create some Open Research Reports on infectious diseases; collections of open publications and datasets brought together using open bibliographic data and crowd-sourced summaries of non-open content. This would be fully searchable and semantically linked and would enable discovery of open research by academics and others, with particular public interest likely to stem from patient groups. Open Research Reports are primarily being developed by David Shotton and Tanya Gray (University of Oxford), and we hope that this project will expand in scope and grow into a valuable resource for the life sciences, fuelled by the increasing availability of open data and content.

Additionally, the working group has several members researching technologies that will use open data to seek new scientific discoveries, which nicely illustrate its potential. In the semantic web community, much effort has been made to link life sciences data together in a way that machines can understand the semantic links between objects in datasets. This will not only assist in keeping track of the rapidly expanding scientific literature, but also will enable novel analyses to be performed and new connections discovered, for example, linked open drug data aims to connect previously unlinked results from clinical trials, gene expression assays, and chemical testing [10]. This enables researchers to more rapidly answer complex queries using a single interface rather than manually searching through the literature; one example would be to discover possible targets of a medicine by searching for the possible targets of drugs with shared ingredients. Drawing together diverse datasets for reuse in this manner becomes complicated where their terms of use are restrictive or not interoperable, making openness a valuable attribute.

The Open Data in Science working group has a common goal of achieving a world in which scientific data is open by default according to the Panton Principles, with limited exceptions. As a diverse collection of individuals, the aims, objectives, and means to achieve this are a matter of healthy debate and we encourage others to join the discussion.

In terms of our primary aim of providing tools, apps, and datasets for generating, discovering, and reusing open data, ideas are flowing continuously but require the input of the wider scientific community in identifying the problems they face in publishing, discovering, and reusing data online and requesting assistance in solving them. The working group aims to provide a community and network that can respond to these needs and a hub for access to the resulting tools, which we hope all stakeholders in scientific data will find valuable. Better science—in terms of transparency, reproducibility, increased efficiency, and ultimately a greater benefit to society—depends on open data.

## Abbreviations

BMC: BioMedCentral
IIOD?: Is It Open Data?
OA: open access
OKD: Open Knowledge Definition
OKF: Open Knowledge Foundation
WDTK?: What Do They Know?
